# Generative Artificial Intelligence (AI) in Medical Education: A Narrative Review of the Challenges and Possibilities for Future Professionalism

**DOI:** 10.7759/cureus.86316

**Published:** 2025-06-18

**Authors:** Nobuyasu Komasawa, Masanao Yokohira

**Affiliations:** 1 Community Medicine Education Promotion Office, Faculty of Medicine, Kagawa University, Miki-cho, JPN; 2 Department of Medical Education, Kagawa University, Miki-cho, JPN

**Keywords:** ai literacy, ethical challenges, generative ai, medical education, medical professionalism

## Abstract

The rapid emergence of generative artificial intelligence (AI) is reshaping the landscape of medical education and healthcare. Unlike traditional AI, which focuses on classification or prediction, generative AI can create novel content-such as clinical notes, patient education materials, and simulated interactions-based on large-scale data. This capacity offers significant opportunities for personalized learning, clinical efficiency, and patient engagement. However, the integration of generative AI also introduces complex challenges, including ethical ambiguity, misinformation, accountability, data privacy risks, and potential erosion of critical thinking skills. These risks are especially salient in educational settings, where future physicians are still developing their professional identities. In this narrative review, we examine the dual role of generative AI as both a transformative tool and a source of ethical and professional disruption. We analyze its benefits and challenges across educational and clinical domains and argue that the traditional model of medical professionalism must evolve in response. Drawing on international literature and diverse cultural contexts in medical education, we propose a redefined framework for AI-era professionalism-one that integrates technological fluency with enduring humanistic values such as empathy, integrity, and accountability. This review offers AI-integrated medical professionalism to prepare future physicians to use generative AI responsibly, ethically, and in service of patient-centered care.

## Introduction and background

The emergence of generative artificial intelligence (AI) is rapidly transforming the landscape of healthcare. Unlike traditional AI systems that focus on classification, prediction, or data retrieval, generative AI possesses the ability to create new content-such as text, images, or code-based on learned patterns from large datasets [1]. This unique capability opens new possibilities across various domains of medicine, including clinical practice, patient communication, and research [2].

Building on this foundational understanding of generative AI's capabilities, we now explore how these technologies are being applied in clinical practice to enhance efficiency, communication, and patient care [3]. Administrative tasks in clinical settings can be streamlined through the automated generation of medical notes, referral letters, and discharge summaries using generative AI [4]. It can assist physicians by summarizing clinical guidelines, generating differential diagnoses, or even composing patient-friendly explanations of medical conditions [5]. Moreover, with multilingual capabilities, generative AI has the potential to improve communication with diverse patient populations and bridge language barriers in global healthcare delivery.

Beyond clinical utility for healthcare providers, generative AI also holds promise in engaging patients directly, fostering greater autonomy and participation in their own health management. Generative AI also plays a valuable role in patient empowerment and chronic disease management [6]. Conversational AI systems can provide lifestyle advice, medication reminders, and mental health support, enabling patients to engage more actively in their care. By generating customized educational content, these systems can improve health literacy and encourage preventive behaviors among the general population [7].

While generative AI empowers both clinicians and patients in practical care settings, it also accelerates scientific discovery by transforming how medical research is conducted [8]. In the realm of research, generative AI facilitates the automation of literature reviews, the drafting of research protocols, and the development of scientific manuscripts. In addition, in fields such as drug discovery, generative models can propose novel molecular structures, accelerating the process of identifying therapeutic candidates. These capabilities significantly enhance productivity and innovation in medical science [9].

However, the rapid adoption of generative AI is not without its challenges-particularly in terms of ethics, accountability, and the safe integration of these tools into healthcare workflows. Despite these advancements, generative AI raises several ethical and professional challenges that must be addressed [10]. One major concern is the risk of misinformation or “hallucinations,” where AI generates plausible but factually incorrect content. For example, large language models may fabricate academic references that appear credible but do not actually exist, leading to misinformation in scholarly or clinical contexts. Additionally, they can suggest implausible differential diagnoses-such as linking pneumonia with endocrine disorders-without clinical justification, potentially misleading learners. In the medical context, such errors could directly impact patient safety, underscoring the need for careful oversight and expert validation of AI-generated information.

Another pressing issue is the question of accountability [11]. When generative AI is involved in clinical documentation or decision support, it is essential to clarify who bears responsibility for the content and any outcomes that result from its use. Physicians must maintain professional responsibility for reviewing and verifying any AI-generated outputs before using them in patient care.

Data privacy is also a critical consideration. Inputting identifiable patient data into generative AI systems-especially those operated by third parties-poses risks unless strict data protection and anonymization protocols are followed [12]. In parallel, there is growing concern that overreliance on AI tools in education and practice may erode critical thinking and clinical reasoning skills among medical professionals [13]. As the use of generative AI becomes more widespread, it is imperative that healthcare systems establish clear guidelines for its ethical and responsible use [1]. Medical education should incorporate AI literacy as a component of professionalism, teaching future healthcare providers not only how to use AI tools but also how to recognize their limitations, interpret their outputs critically, and uphold the principles of patient-centered care.

In summary, generative AI holds immense potential to enhance the quality, efficiency, and accessibility of healthcare. However, its successful integration requires a careful balance between technological advancement and humanistic values. As the profession evolves, medical professionals must remain committed to core principles-such as empathy, ethical responsibility, transparency, and accountability-while leveraging AI as a supportive tool [14]. Generative AI should not replace the clinician but rather empower them to deliver more informed, compassionate, and efficient care.

In this narrative review, we aim to analyze the dual role of generative AI in medical education and healthcare-its transformative potential and its ethical challenges-and to propose a redefined model of professionalism that aligns with cultural and institutional context.

## Review

Benefits of generative AI in medical education and healthcare

Generative AI has emerged as a transformative force in medical education and healthcare, offering novel capabilities that go beyond traditional AI systems. Unlike conventional algorithms that are limited to classification or prediction tasks, generative AI can produce original content-ranging from textual explanations and visual simulations to synthetic patient interactions [15]. These capacities significantly expand the potential for innovation in medical training and practice.

One of the most promising advantages of generative AI is the facilitation of personalized learning. By adapting to individual learners’ levels, preferences, and progress, AI can generate tailored quizzes, simplified explanations of complex topics, and customized study plans [16]. These tools act as responsive, on-demand tutors, providing real-time feedback and promoting greater engagement. Students can repeatedly engage with virtual patient scenarios and improve their diagnostic and communication skills through interactions with AI-generated personas [17]. Such simulations not only enhance knowledge retention but also help learners build confidence in clinical decision-making and interpersonal communication. However, despite these advantages, the accuracy and appropriateness of AI-generated personalized content remain dependent on the quality of training data and prompt engineering. Current models lack a robust mechanism to validate the pedagogical effectiveness or factual reliability of individualized content across diverse learner populations. Moreover, there is limited empirical evidence to confirm that AI-driven personalization consistently leads to improved educational outcomes, highlighting the need for rigorous validation studies and ongoing oversight by human educators.

Generative AI also offers substantial support for scholarly and academic activities. Students and researchers can utilize AI tools to summarize research articles, draft scientific abstracts, and generate visual aids for presentations [18]. For educators, these tools reduce the administrative and cognitive burden of designing curricula, formulating examination items, and grading, thereby allowing more time for mentorship and interactive teaching. Additionally, generative AI can assist in identifying knowledge gaps among students, enabling instructors to provide more targeted instruction.

Furthermore, clinical training benefits significantly from AI-driven simulations and case-based learning environments. These platforms enable learners to experience a wide variety of medical scenarios-some of which may be rare in real clinical settings. The ability to receive immediate, adaptive feedback fosters experiential learning and supports the development of critical clinical competencies [19,20]. For instance, virtual scenarios involving difficult patient interactions or rare disease presentations can help students gain exposure to complex ethical and diagnostic challenges.

Challenges of generative AI in medical education and healthcare

While the benefits of generative AI are substantial, its integration into healthcare and education also raises a host of ethical, cognitive, and systemic concerns [21,22]. Understanding these challenges is essential for ensuring the responsible use of AI in medical settings.

Despite the remarkable benefits, generative AI introduces several critical challenges that must be addressed in medical education. Among the foremost concerns is the issue of factual accuracy. AI-generated content, while often convincing in style, may contain significant errors or misleading statements if not properly verified [23]. Students who rely uncritically on such outputs risk internalizing incorrect medical information, potentially jeopardizing future clinical competence and patient safety.

Another concern is the possible erosion of core cognitive and ethical skills. Overreliance on AI tools may hinder the development of essential abilities such as clinical reasoning, diagnostic acumen, and ethical decision-making [24]. These skills are fundamental to the medical profession and require deliberate cultivation through problem-solving, reflection, and guided practice. Generative AI, if used without restraint, may inadvertently undermine these developmental processes and contribute to superficial learning.

The risk of academic dishonesty also grows with increased access to generative tools. Students might use AI to write essays, generate assignments, or even fabricate research data, raising complex issues related to authorship, originality, and integrity [25]. In the absence of clear institutional guidelines on AI usage and citation, these behaviors may go unchecked, leading to an erosion of academic standards and compromising the credibility of educational institutions.

Equity is another pressing ethical issue. High-quality generative AI tools often require robust digital infrastructure and financial investment. Disparities in access could exacerbate educational inequalities between institutions or geographic regions, further marginalizing students in under-resourced environments [26]. This digital divide not only impacts learning outcomes but also challenges the fairness and inclusivity of AI-integrated medical education.

To mitigate these risks, it is essential to incorporate structured AI literacy education into medical training. Students must be equipped not only with the technical skills to effectively use generative AI but also with critical thinking abilities to assess its outputs, identify inaccuracies, and understand the ethical implications of its use. A hybrid educational model, in which human educators collaborate with AI systems, appears most effective. In this approach, AI functions as an assistant to personalize and optimize instruction, while educators provide necessary contextualization and emotional support and serve as professional role models [18]. Additionally, institutions need to develop clear policies regarding the ethical use of generative AI to ensure that its adoption does not undermine educational integrity, academic rigor, or patient safety.

Current state and vulnerabilities of professionalism in medical education in the AI era

Ultimately, the adoption of generative AI in medical education should be grounded in the enduring values of medical professionalism. While AI can enhance efficiency and accessibility, it must not replace the ethical judgment, empathy, and responsibility that define the medical profession [22]. Instead, it should be seen as a powerful supplement-one that, when thoughtfully integrated, can help train physicians who are both technologically fluent and deeply humanistic.

Medical professionalism has long been a cornerstone of medical education, reflecting values such as integrity, accountability, respect, and compassion. However, the rapid integration of AI technologies into healthcare and education presents new challenges that threaten to undermine these foundational principles [27].

The formal teaching of professionalism has traditionally been supplemented by an implicit “hidden curriculum” conveyed through clinical apprenticeships and role modeling [28]. Yet, with the increasing presence of AI, there is a risk that the emphasis on interpersonal interactions, ethical reflection, and human-centered care may be diminished [29]. As medical students become more reliant on AI for learning and decision-making, the tacit transmission of professional values may weaken, and the moral development of students may become increasingly fragmented.

One emerging vulnerability is the ambiguity of accountability in clinical settings that use AI. When diagnostic or therapeutic recommendations are generated by algorithms, it becomes unclear who bears ultimate responsibility for medical decisions-the clinician, the developer, or the institution [30]. This ambiguity is particularly concerning in an educational context, where students are still developing their ethical frameworks and professional identities. Another issue is transparency. Many generative AI systems operate as black boxes, offering outputs without revealing the logic or evidence behind them [31]. This opacity challenges the traditional approach to medical communication, which values clarity, empathy, and shared decision-making [29]. If students are not trained to question and interpret AI outputs, their ability to engage in ethical clinical dialogue and explain decisions to patients may erode.

Moreover, medical education faces cultural and structural barriers in integrating professionalism training that explicitly addresses the challenges of AI. Hierarchical teaching methods, limited curricular flexibility, and insufficient faculty development hinder the timely adaptation of professionalism education to technological changes [32]. Without urgent reform, these factors may leave future physicians ill-prepared to navigate the ethical complexities introduced by AI and to maintain the trust placed in them by society. To assess such AI-related competencies, structured approaches such as Objective Structured Clinical Examination (OSCE) and professional portfolios can offer partial but meaningful insights into learners’ ability to critically evaluate and ethically apply AI tools. However, further research and curricular innovation are needed to establish valid and reliable methods for evaluating these emerging dimensions of medical professionalism.

A proposal for a new era of professionalism for AI-augmented physicians

As traditional models of professionalism face growing strain in the face of rapid technological change, it has become increasingly necessary to reconsider and redefine what constitutes professionalism in the era of AI-driven healthcare. In this section, we outline a revised framework that reflects the evolving demands of AI-augmented clinical practice.

**Figure 1:**
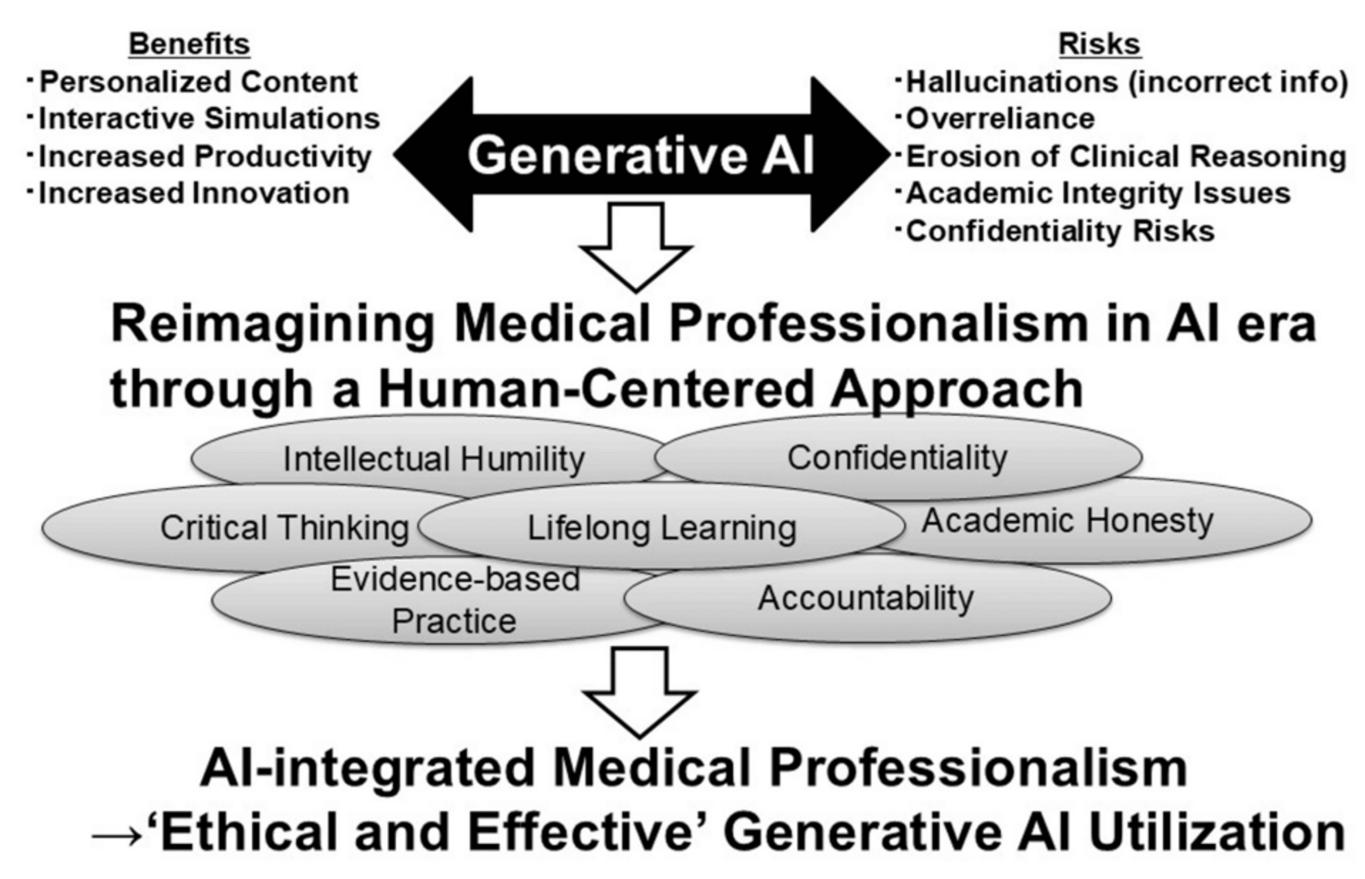
AI-Integrated Medical Professionalism Framework for Effective Generative AI Utilization. Created by the authors AI: artificial intelligence

Given these evolving challenges, it is imperative to reconceptualize medical professionalism for the AI era and to redesign educational strategies accordingly [33,34]. The emergence of generative AI does not diminish the need for professionalism-it amplifies it. A new framework is required, one that integrates technological fluency with enduring human values and prepares future physicians to be both competent clinicians and ethical stewards of AI. This updated professionalism must emphasize intellectual humility, critical thinking, ethical discernment, and a strong sense of accountability (Figure 1).

Students should be encouraged to view AI not as a replacement for human judgment but as a tool to be used responsibly within the context of patient-centered care [35]. For example, when an AI system suggests a diagnosis or treatment plan, it is the physician’s responsibility to evaluate the recommendation, consider the patient’s values and context, and make the final decision.

Medical curricula should be revised to include structured modules on AI ethics, data privacy, algorithmic bias, and digital communication [36]. Reflective writing, case-based discussions, and simulation exercises involving AI tools can help students explore these issues in depth [37]. Interdisciplinary courses that include engineering, law, and ethics may also offer valuable perspectives [38]. Faculty must also receive training to model this new professionalism, demonstrating how to integrate AI into clinical practice with transparency, empathy, and ethical rigor.

Importantly, this transformation must be culturally contextualized. In certain cultural or institutional contexts where hierarchical structures and implicit norms strongly shape medical training, it is particularly important for educators to encourage open dialogue and reflective practice. Such efforts enable students to critically examine the evolving role of AI in medicine within their specific sociocultural settings [39]. Interdisciplinary collaboration with ethicists, engineers, and patients can further enrich this process and promote a more holistic understanding of professionalism in the digital age.

The integration of generative AI into medical education necessitates a renewed commitment to professionalism [40,41]. By embracing AI-integrated medical professionalism-which includes AI literacy, ethical responsibility, and humanistic care-medical education can produce physicians who are not only skilled in using advanced technologies but also deeply committed to the values that sustain patient trust. Such a vision ensures that as AI becomes more prominent in healthcare, it does so under the guidance of compassionate, competent, and principled human professionals.

## Conclusions

The integration of generative AI into medical education and healthcare is transforming the landscape of learning, clinical training, and professional identity. Its capacity to deliver personalized, scalable, and interactive educational experiences presents unprecedented opportunities for enhancing medical competence. However, the power of generative AI also introduces significant challenges-ranging from factual inaccuracies and overreliance to ethical ambiguity, academic dishonesty, and widening educational inequalities. These risks, if unaddressed, could undermine the core values of medicine and the integrity of future clinical practice. The hidden curriculum alone is no longer sufficient to foster the ethical discernment and accountability needed in a digital healthcare environment. Moreover, traditional hierarchies and rigid curricular structures pose additional barriers to adapting quickly to technological change.

To navigate this transition responsibly, medical education must reconceptualize professionalism for the AI era. This involves not only technical AI literacy but also a deepened commitment to humanistic care, ethical reasoning, and critical reflection. AI should be viewed not as a surrogate for clinical judgment but as a partner that supports informed, patient-centered decisions. Faculty development, interdisciplinary collaboration, and culturally sensitive pedagogical reform are essential for building this new model of professionalism. Ultimately, the future of medicine lies in harmonizing technological advancement with timeless professional values. Even with structural reforms in place, unchecked reliance on AI may, over time, subtly erode the core values of medical professionalism without practitioners fully realizing it. By training physicians who are both technologically adept and ethically grounded, we can ensure that generative AI serves as a force for good-enhancing, rather than eroding, the trust, empathy, and integrity that define the medical profession.
